# Multiple Biventricular Intracardiac Thrombi—An Unusual Finding in Peripartum Cardiomyopathy: A Case Report

**DOI:** 10.1002/ccr3.71014

**Published:** 2025-09-28

**Authors:** Gidion Edwin, Yohana Mbishi, Baraka Alphonce, John Meda

**Affiliations:** ^1^ Department of Internal Medicine, School of Medicine and Dentistry The University of Dodoma Dodoma Tanzania; ^2^ Department of Internal Medicine The Benjamin Mkapa Hospital Dodoma Tanzania; ^3^ Department of Cardiology The Benjamin Mkapa Hospital Dodoma Tanzania

**Keywords:** anticoagulation therapy, biventricular intracardiac thrombi, heart failure, peripartum cardiomyopathy

## Abstract

Peripartum cardiomyopathy (PPCM) is a rare form of heart failure occurring during late pregnancy or within 5 months postpartum, its etiology remaining elusive. While typically characterized by left ventricular dysfunction, it may present with intracardiac thrombi, raising the risk of systemic embolization. Here, we report an unusual case of PPCM featuring biventricular intracardiac thrombi, highlighting the importance of a thorough investigation for potential underlying causes. This is a case of a black African, multiparous woman aged 44 years with an unremarkable past medical history, who presented with symptoms suggestive of acute heart failure 5 months after her last delivery. A 2D Transthoracic Echocardiography showed multiple biventricular intracardiac thrombi, a rare complication of PPCM without a systemic embolic event. Further, laboratory tests for evidence of a hypercoagulable state came out negative. Treatment involved a combination of acute heart failure and anticoagulation followed by optimal medical therapy for chronic heart failure, resulting in improved symptoms for heart failure, cardiac function, and significant thrombi resolution after 8 weeks. Multiple biventricular intracardiac thrombi are an uncommon finding in PPCM, underscoring the importance of vigilant diagnosis and timely management. Although the management of PPCM remains challenging due to limited evidence, an individualized approach to heart failure therapy and anticoagulation can lead to meaningful clinical improvement and favorable outcomes.

**Summary**

- Biventricular intracardiac thrombi are a rare peripartum cardiomyopathy consequence.
- Echocardiography is essential for early diagnosis and prompt treatment.
- Complete thrombus resolution may result from prompt anticoagulation.
- Overall cardiac function and results are improved with individualized heart failure treatment.

## 1 Introduction

Peripartum cardiomyopathy (PPCM) is a cause of heart failure that occurs in the last months of pregnancy and within 5 months after delivery in a previously healthy woman [1]. It is characterized by left ventricular dysfunction and dilatation; however, its cause remains unknown [2]. Its incidence is 1 per 3000 to 1 per 4000 live births [2, 3]. The incidence of PPCM has displayed geographical variability whereby some places like Japan have the lowest of 1 in 20,000 live births. In the United States, it is 1 in 4000, and the highest in Nigeria is 1 in 100 live births with a fatality rate of between 20% and 50% [3, 4].

Intracardiac thrombus is commonly encountered among patients with PPCM, its prevalence ranges between 10% and 17% [5, 6, 7]. However, biventricular intracardiac thrombi are an uncommon presentation [8]. The presence of intracardiac thrombi increases the risk of systemic embolization [6]. The underlying mechanisms for intracardiac thrombogenesis that have been postulated include complications of blood stasis, hematological hypercoagulability, inflammatory conditions, cardiomyopathic states, or genetic conditions and deficiencies [4, 5, 9].

Most intra‐cardiac thrombi involve the left‐sided cardiac chambers; involvement of the right side is a rare entity, and there is rising suspicion of finding a secondary cause. Here we present a case of PPCM with biventricular intracardiac thrombi without systemic embolization or a secondary cause.

## 2 Case Presentation

### 2.1 Demographics and Medical History

Here, we present a case of a black African multiparous woman aged 44 years with an unremarkable past medical history. She is parous 6 with 6 living children; all previous pregnancies were uneventful. Her first delivery was 22 years ago, and her last delivery was 5 months ago before the index presentation. She was referred from a health facility in the upcountry with a diagnosis of acute heart failure secondary to PPCM. She presented with progressive dyspnoea on exertion, dry cough, paroxysmal nocturnal dyspnoea, orthopnoea, and anasarca. However, she dismissed a history of chest pain, hemoptysis, immobility, recent surgeries, fever, or contact with the patient confirmed with pulmonary tuberculosis. No symptoms suggest haematuria, diaphoresis, altered mentation/loss of consciousness, or neurological deficits. Besides, she neither smokes nor takes alcohol, and there was no history of preceding chronic diseases like hypertension, diabetes mellitus, or HIV disease.

### 2.2 Clinical Findings

Middle‐aged lady, restless, in respiratory distress, dyspneic, no lymphadenopathy, with anasarca. Her vital signs were: temperature (*T* = 36.8°C), blood pressure 112/61 mmHg, a respiratory rate of 23 cycles/min, and oxygen saturation was 90% in room air. On systemic examination, abnormal findings were confined to the cardiovascular system: raised jugular venous pressure (11.5 cm), diffuse apex beat with S_**3**_ gallop rhythm, and diffuse bibasilar fine crackles, which are in keeping with dilated cardiomyopathy.

### 2.3 Investigations

**FIGURE 1:**
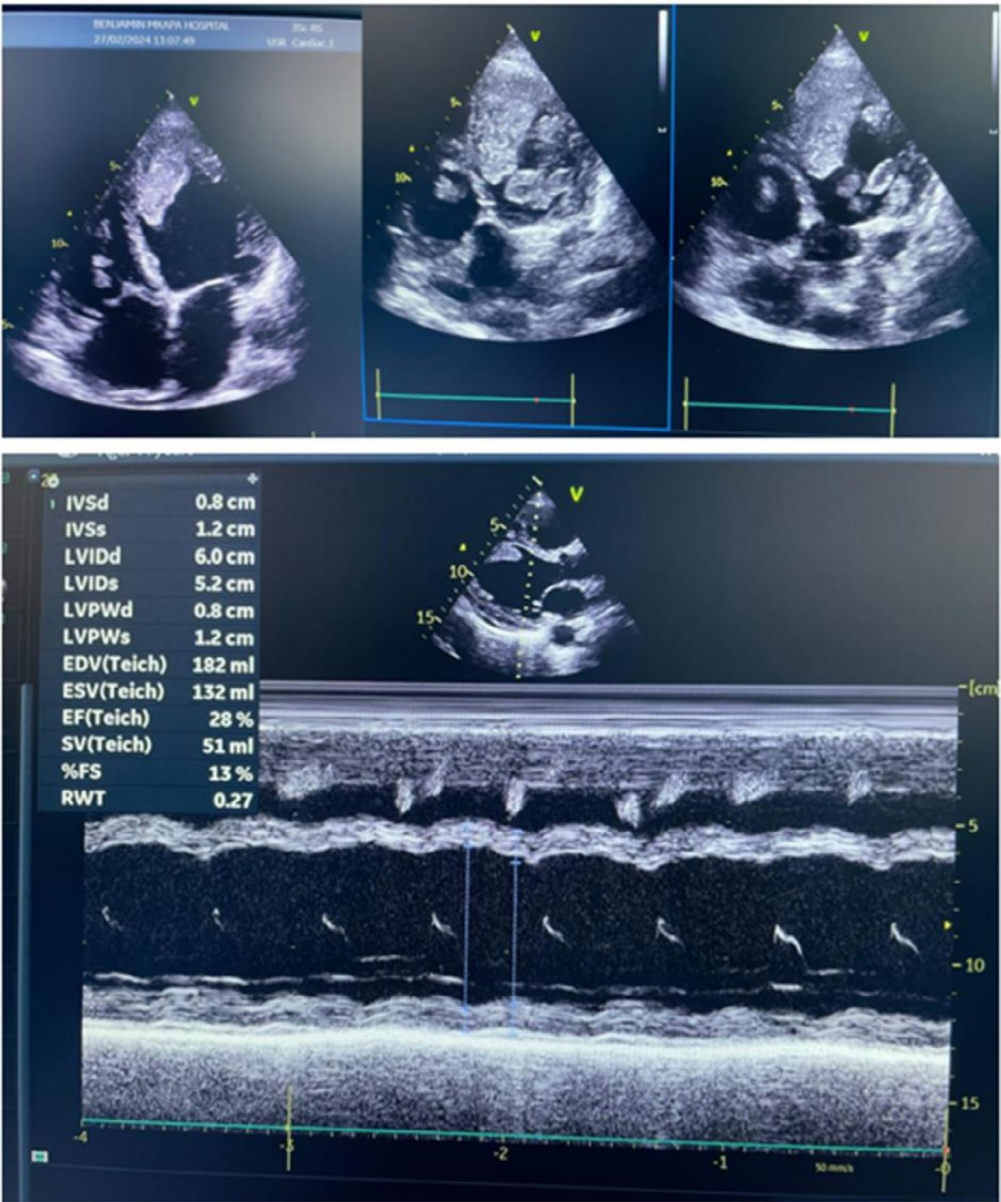
2D 4‐chambers view showing multiple intracardiac thrombi on both ventricles. 2D‐parasternal long axis view M‐mode showing EF of 28% (during admission).

Chest x‐ray showed cardiomegaly with specific features suggestive of pulmonary edema; no effusion was seen. A 12‐lead Electrocardiogram tracing showed sinus tachycardia, biatrial enlargement, low voltage, and non‐specific ST‐T wave changes, consistent with dilated cardiomyopathy. A 2‐dimensional and M‐mode Transthoracic echocardiogram (2D‐TTE) demonstrated global hypokinesia, dilated LV, and RV (bi‐ventricular dilation), and no LV apical aneurysm was seen. Severely impaired LV systolic function with an Ejection fraction (EF) of 28%; impaired RV function with TAPSE of 1.4 cm; dilated mitral and tricuspid annuli with moderate functional MR and TR. All other valves appeared normal in structure and function. No intracardiac shunts were seen. There were multiple intracardiac thrombi seen in LV and RV measuring 1.3 cm. The pericardium was normal with no pericardial effusion; dilated IVC (2.2 cm) non‐collapsing with inspiration; these features are in keeping with dilated cardiomyopathy with multiple biventricular intracardiac thrombi (Figure 1).

Further laboratory tests including tests for the hypercoagulable state are within the normal limits except for D‐dimer, liver enzymes, cardiac enzymes, serum creatinine, and brain natriuretic peptide (Table 1). The serology test for human immunodeficiency virus (HIV) was non‐reactive. Ultimately, the final diagnosis of PPCM with multiple biventricular intracardiac thrombi was established.

**TABLE 1:** Laboratory results.

S/no	Test(s)	Reference value	Result(s)	Remark(s)
1.	High‐sensitive C‐reactive protein	0.0–1.0 mg/dL	2.1	Elevated
2.	Brain natriuretic peptide (BNP)	≤ 100 pg/mL	318.7	Elevated
3.	Prothrombin time (PT)/INR	0.8–1.1 s	1.9	Elevated
4.	Activated partial thromboplastin time (aPTT)	25–40 s	53	Elevated
5.	Serum creatinine level	61–112 mmol/L	89	Normal
6.	Blood urea nitrogen (BUN) level	2.8–7.1 mmol/L	5.6	Normal
7.	Protein C level	63–135 IU/dL	71	Normal
8.	Protein S level	60–150%	84	Normal
9.	Anti‐nuclear antibodies	≤ 1:80	1:58	Normal
10.	Anti‐cardiolipin antibodies (IgM and IgG)	IgM: ≤ 12.5 U/mL	9.1	Normal
		IgG: ≤ 15 U/mL	8.4	Normal
11.	Aspartate transaminase (AST)	8–42 U/L	37.1	Normal
12.	Alanine transaminase (ALT)	8–40 U/L	23.9	Normal

### 2.4 Therapeutic Intervention and Outcomes

Acute heart failure therapy with intravenous furosemide 60 mg thrice daily, and isosorbide mononitrate 10 mg twice daily was initiated. She was also anticoagulated with intravenous unfractionated heparin (UFH) 5000 units twice daily and oral warfarin 5 mg daily for the biventricular thrombi. The target INR range was set between 2.0 and 3.0. Seventy‐two hours after initiating dual anticoagulation therapy, the patient's INR reached 2.3. A marked improvement in acute heart failure symptoms was observed during this period. Given the patient's preference and the logistical challenges of routine INR monitoring in her remote setting, a direct oral anticoagulant (DOAC) was chosen for continued anticoagulation, despite the limited evidence supporting DOAC use in PPCM with intracardiac thrombi. Subsequently, therapy with oral furosemide 40 mg once daily, oral enalapril 5 mg once daily, oral spironolactone 25 mg once daily, oral bisoprolol 5 mg once daily, and oral rivaroxaban 15 mg once daily was continued, and her condition improved significantly. Vericiguat was not initiated in this case due to the patient's favorable response to initial GDMT and the limited evidence for its use in PPCM. Serial echocardiogram on day 6 showcased remarkable findings with reduction of LV and RV thrombi size with LVEF of 34%. The patient continued faring well and was discharged at her request 10 days after admission. The patient decided not to breastfeed because she was afraid for her baby's health due to the adverse effects of pharmacotherapy in PPCM.

**FIGURE 2:**
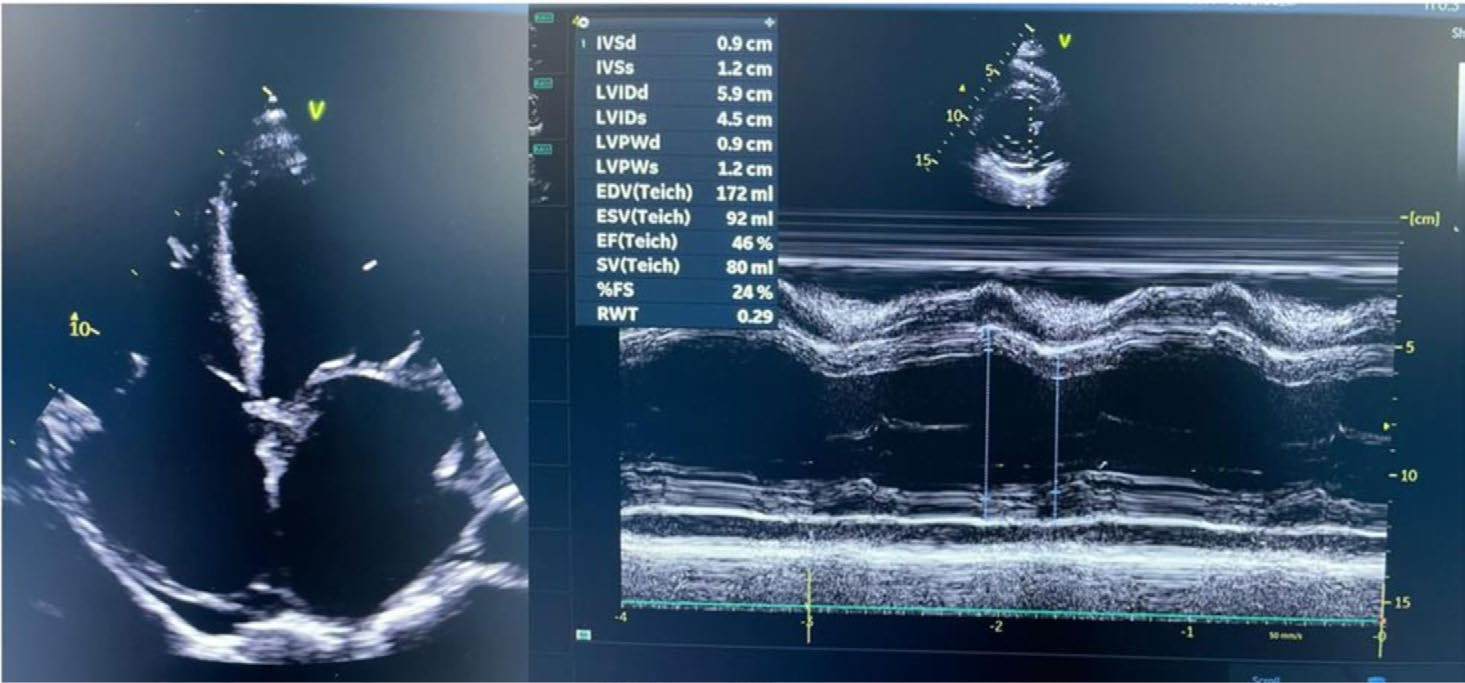
2D 4‐chambers view showing no intracardiac thrombi; 2D PLAX M‐mode showing EF of 46% with marked improved contractility (after 8 weeks of treatment).

Eight weeks later, she attended an outpatient clinic for follow‐up. She was well compensated with a marked resolution of symptoms. She also underwent serial TTE, which showed no evidence of thrombi, and the left ventricle ejection fraction improved to 46% (Figure 2). Currently, she is on the management of chronic heart failure, including, among others, mineralocorticoid receptor antagonist (oral spironolactone), oral loop diuretic (furosemide), and angiotensin‐converting enzyme inhibitors (oral enalapril). An intra‐uterine contraceptive device (IUCD) was inserted for family planning since hormonal‐based contraception was associated with risks for thromboembolic events.

## 3 Discussion

PPCM has been linked to increased rates of thromboembolism when compared to other types of cardiomyopathies. The hypercoagulable state during pregnancy is caused by several factors, such as immobility, endothelial damage, cardiac dilatation, and increased clotting factor production [10].

Biventricular thrombi associated with PPCM are quite rare [11]. To date, only 11 cases have been reported (Table 2). In these reports, the age at presentation ranged between 19 and 39 years [1, 15]; only five cases documented the parity at presentation, which was between 2 and 7 [3, 4, 14, 17, 18], and the onset of heart failure symptoms varied from 2 weeks to 5 months [3, 13]. In the index case, our patient was in her early 40s; she was quite older compared to patients in previous cases. Her gravidity was 6, and the manifestations of heart failure symptoms began 5 months post‐delivery, similar to existing cases.

**TABLE 2:** Descriptive summary of case reports (1–11) describing biventricular intracardiac thrombi in patients with PPCM.

Study, year	Age (years)	Parity	Presentation postpartum	Ejection fraction (%)	Number of thrombus	Embolic events	Anticoagulant used	Time to thrombus disappearance
Bhat et al., 1986 [12]	20	N/A	4 months	39	Biventricular thrombi	Saddle pulmonary embolism and bilateral femoral artery embolism	Unfractionated heparin and warfarin	N/A
Nishi et al., 2002 [13]	23	N/A	2 months	18	Apical thrombi, freely mobile, measuring 1 cm within both ventricles	No evidence	Warfarin	4 days
Ibebuogu et al., 2007 [14]	24	5	5 months	15	Single Thrombi on each ventricle	Splenic, hepatic, and renal	Low molecular weight heparin	5 days
Sánchez‐Rubio Lezcano et al., 2004 [15]	39	N/A	4 months	28	Large thrombus occupying the apex of the right ventricle and another thrombus adhered to the septum of the left ventricle	No evidence	Low molecular weight heparin	N/A
Koç et al., 2011 [16]	21	N/A	2 months	35	Left ventricular apical thrombus was oval, fresh, and mobile; the thrombus at the right ventricular apex was spherical, fresh, and mobile	Cerebral embolism	Unfractionated heparin	12 days
Kim et al., 2011 [11]	22	N/A	4 months	17	Larger in the right ventricle, smaller in the left ventricle	No evidence	Warfarin and unfractionated heparin	16 days
Sakamoto et al., 2014 [4]	37	2	4 months	29	Left ventricular thrombus (2.8 × 2.1 cm, 2.2 × 1.4 cm) and right ventricular apex (1.6 × 1.1 cm)	Cerebral embolism	Warfarin and unfractionated heparin	10 days
Acar et al., 2017 [1]	19	N/A	3 months	20	Multiple thrombus in left ventricle and right ventricle	No evidence	Unfractionated heparin	N/A
Abi Jaoude et al., 2023 [3]	32	7	5 months	15–20	Biventricular fixed thrombi	Hepatic	Apixaban	N/A
Gunes, 2018 [17]	22	4	2 weeks	30	Two oval‐shaped thrombi in the left ventricle and a single thrombus in the right ventricle	Pulmonary embolism	Warfarin and unfractionated heparin	N/A
Al Habsi and Al Lawati, 2022 [18]	29	2	2 weeks	10–15	Large thrombi occupying the left ventricle measuring (2.3 × 1.9 cm) and right ventricle measuring (2.6 × 4.5 cm)	No evidence	Warfarin and low molecular weight heparin	3 months
Menashe et al., 2021 [19]	33	N/A	3 months	10	Multiple left ventricular and large right ventricular	Pulmonary embolism	Unfractionated heparin	N/A

Among the cases reported, the ejection fraction was less than or equal to 20% in 6 cases, the lowest reported ejection fraction was 10% and the highest was 39% [12, 19]. In the index case, the ejection fraction was 28% similar to existing cases. Embolic events were common in reported cases; only 5 cases didn't show evidence of embolic phenomena [1, 11, 13, 15, 18] similar to the index case. Among the cases that demonstrated embolic events: pulmonary embolism was reported in 2 cases, cerebral embolism in 2 cases, and hepatic infarction in 2 cases. Among the cases with hepatic embolism, there were embolic events involving the spleen and kidney [4, 12, 14, 17, 19].

There is no consensus or clinical guidelines to outline the best management strategy for ventricular thrombus due to PPCM [20]. According to the American Heart Association (AHA) guideline in 2013, anticoagulation is recommended for patients with PPCM if left ventricular ejection fraction (LVEF) is < 30%. Whereas, the guidelines from the European Society of Cardiology (ESC) 2018 recommend anticoagulation in women with PPCM with LVEF ≤ 35% (level of evidence IIB) [6]. A previous review of anticoagulation uses for intracardiac thrombi in PPCM recommended the use of either Heparin (either unfractionated heparin or low molecular weight heparin) or warfarin, which is typically recommended in postpartum women when there are clear indications such as left ventricular thrombi, severely reduced left ventricular ejection fraction (LVEF < 30%), atrial fibrillation, or systemic thromboembolism [20, 21]. In our case, the patient met the criteria for anticoagulation with warfarin due to the presence of intracardiac thrombi. However, given the logistical challenges associated with routine INR monitoring in her remote residence, the patient opted for a DOAC as a more practical long‐term alternative [20]. Similarly, among the cases of biventricular thrombi reported, heparin and warfarin were commonly used, except in a single case where the directing acting anticoagulant (DOAC) was used. In our case, we used warfarin and heparin, but later on, she was maintained on DOAC. The duration of anticoagulation is another ambiguous topic. As per the heart failure guidelines for PPCM, anticoagulation therapy should be continued until left ventricular function normalizes [2], that's within 2 to 6 months in 23% to 72% of cases, but it can take longer, up to 5 years [22]. In previous cases of biventricular thrombi, 5 patients had clot resolution within 3 weeks [4, 11, 13, 14], except a single patient who had clot resolution after 3 months [18], and 5 other cases lacked data on clot resolution [1, 3, 12, 15, 17, 19]. Similarly, the time to clot resolution in our case was 2 weeks. There was a linear relationship between thrombus burden or size and time to thrombus resolution; as the number or size of thrombus increased, the time to thrombus resolution was prolonged. The use of mineralocorticoid receptor antagonists such as spironolactone, along with beta‐blockers and angiotensin‐converting enzyme (ACE) inhibitors or angiotensin receptor blockers (ARBs), is indicated in the management of PPCM and has been associated with improved survival outcomes by reducing cardiovascular mortality [21, 23]. Bromocriptine has also been proposed as an adjunct therapy in PPCM due to its ability to suppress prolactin, a hormone implicated in disease pathogenesis [20, 21]. However, its use is limited by concerns regarding thromboembolic complications, and data on its safety and efficacy remain limited. Pharmacotherapy in the postpartum period must be approached with caution, especially in breastfeeding women. Several heart failure medications, including beta‐blockers, ACE inhibitors, and diuretics, may be excreted in breast milk and could potentially cause adverse effects in infants—such as bradycardia, hypotension, fluid loss, and electrolyte imbalances [20]. While literature suggests that breastfeeding may be a secondary consideration in the acute management of PPCM, intensive diuresis commonly used in acute heart failure can significantly reduce milk production [20, 23]. Moreover, because prolactin has been implicated in adverse myocardial remodeling, including fibrosis, breastfeeding is often discouraged in favor of prioritizing maternal recovery.

## 4 Conclusion

Multiple biventricular intracardiac thrombi are an uncommon finding in PPCM, underscoring the importance of vigilant diagnosis and timely management. Although the management of PPCM remains challenging due to limited evidence, an individualized approach to heart failure therapy and anticoagulation can lead to meaningful clinical improvement and favorable outcomes.

## Author Contributions

**Gidion Edwin:** writing – original draft, writing – review and editing. **Yohana Mbishi:** writing – original draft, writing – review and editing. **Baraka Alphonce:** writing – review and editing. **John Meda:** supervision, writing – review and editing.

## Ethics Statement

Ethical clearance was not necessary for this case report. Therefore, in this particular case, this section is not applicable.

## Consent

In compliance with the journal's patient consent policy, written informed consent was obtained from the patient in order to publish this case report.

## Conflicts of Interest

The authors declare no conflicts of interest.

## Data Availability

This case report's complete data is available on a justifiable request.
